# Diagnostic Value of Serum Sodium Level and Neutrophil-to-Lymphocyte Ratio in Predicting Severity of Acute Appendicitis: A Retrospective Cross-Sectional Two-Center Study

**DOI:** 10.3390/medicina60111844

**Published:** 2024-11-09

**Authors:** Serdar Senol, Mustafa Kusak, Dursun Burak Özdemir, Ahmet Murat Sendil

**Affiliations:** 1 Department of Surgical Gastroenterology, Samsun Training and Research Hospital, 55090 Samsun, Turkey; 2 Department of General Surgery, Samsun Training and Research Hospital, 55090 Samsun, Turkey; mkusak75@yahoo.com; 3 Department of Surgical Oncology, Faculty of Medicine, Eskişehir Osmangazi University, 26040 Eskişehir, Turkey; dursun_burak@yahoo.com; 4 Department of General Surgery, Faculty of Medicine, Eskişehir Osmangazi University, 26040 Eskişehir, Turkey; ahmetmuratsendil@gmail.com

**Keywords:** appendectomy, appendicitis, biomarkers, disease severity, predictivity

## Abstract

*Background and Objectives*: The best way to distinguish complicated acute appendicitis (CAA) from uncomplicated acute appendicitis (UCAA) is still under debate. The aim of this study was to investigate the potential use of the serum sodium (Na^+^) level and the neutrophil-to-lymphocyte ratio (NLR) to distinguish CAA from UCAA and to evaluate whether CAA is more reliably diagnosed using these two variables together. *Materials and Methods*: This was a retrospective, cross-sectional, two-center study of patients diagnosed with AA between 1 January 2016 and 31 December 2023. The demographic and analytical variables were analyzed. The NLR was defined as the quotient between the absolute values of neutrophils and lymphocytes. Hyponatremia was defined as a serum Na^+^ level of ≤135 mmol/L. The sensitivity and specificity of the NLR and the serum Na^+^ level in the diagnosis of CAA were determined by assessing the receiver operating characteristic curves. *Results*: Among the patients who underwent an appendectomy, 3066 histologically confirmed AA cases were identified and included in this study. The registered data revealed that 348 (11.3%) patients had CAA, and the remaining 2718 (88.7%) patients had UCAA. The mean ages were 49.47 ± 18.97 and 38.16 ± 14.50, respectively (*p* < 0.001). The analysis revealed an exponential correlation between the NLR and the serum Na^+^ level with a moderate degree of agreement with CAA (Cohen’s Kappa: 0.461, *p* < 0.001). For CAA, using the NLR and the serum Na^+^ level, the areas under the curve and the cutoffs were 0.664, 4.2 with a confidence interval (CI) of 0.647–0.681 and 0.727, 135 mmol/L with a CI of 0.711–0.742, respectively; all these values were significant with a *p*-value of <0.001. *Conclusions*: Although the serum Na^+^ level is a more effective marker than the NLR, using these two variables together can help detect high-risk patients who may benefit from early management by limiting delays in surgery.

## 1. Introduction

Acute appendicitis (AA) is a major cause of intraabdominal infectious diseases [1], and appendectomy is the most common management approach applied in general surgery clinics [2]. The estimated lifetime risk is 23 percent in females and 12 percent in males [2]. Although this condition is considered to be more common among young patients, it frequently affects the older adult population as well [3]. The diagnosis depends on patient records, a physical examination, and laboratory and radiological investigations [1,4]. Different prediction models are available to improve diagnosis [1,5,6,7,8]. The most well-known, widely used methods are the Alvarado and appendicitis inflammatory scores [5,6].

Surgery has been the standard treatment for over a century. More recently, non-operative treatment with antibiotics has emerged in cases that do not progress to perforation, gangrene, or abscess formation [1,4,9]. On the other hand, complicated cases should be treated surgically because delays may result in complications and a prolonged recovery period [1,10]. However, none of the prediction models can differentiate CAA and UCAA [5,6]. Imaging modalities are usually used to confirm the diagnosis of AA. Additionally, they can be used to guide the differentiation of CAA and UCAA. Although computed tomography (CT) is the preferred radiological imaging technique for this purpose, availability is determined by the local conditions, and ionizing radiation has potentially harmful effects, especially in pregnant people and children, which may limit its broad application [11,12]. In addition to these limitations, according to a recent systematic review, no imaging modality can accurately exclude complicated cases [11].

Complete blood counts and electrolytes are laboratory parameters that are always measured in emergency departments. Furthermore, white blood cell, hemoglobin, platelet, neutrophil, lymphocyte, sodium, potassium, chlorine, urea, and creatinine levels are indispensable for initial assessments and subsequent investigations. The diagnosis of AA and distinguishing complicated cases by using blood markers has been a topic of interest in numerous studies. Some of these studies revealed that an elevated white blood cell count is a common finding among patients with CAA, but its predictive value in differentiating complicated cases from non-complicated ones is low [13,14]. In spite of the fact that initial hospital admission records do not always include blood bilirubin levels, some retrospective studies based on small populations mentioned that hyperbilirubinemia may be a prognostic factor to support the diagnosis of CAA [15,16,17]. On the other hand, a meta-analysis of the precision of hyperbilirubinemia in diagnosing CAA concluded that the diagnostic efficacy of this variable is low [18]. The serum sodium (Na^+^) level and the neutrophil-to-lymphocyte ratio (NLR) are two different topics on this subject. Both of them are easily accessible and interpretable blood markers. The serum Na^+^ level decreases due to non-osmotic vasopressin secretion caused by a series of events initiated by the outer membrane components of Gram-negative bacteria and mediated by the release of proinflammatory cytokines [19,20,21,22]. An increase in the NLR, elevated neutrophil contents, and decreased lymphocyte levels are also caused by the same events [19,20]. They may reflect the severity of inflammation as possible negative and positive acute-phase reactants. The diagnostic role of hyponatremia for CAA, especially in children, has recently been validated [22,23,24,25,26,27]. Similarly, recent studies have investigated the accuracy of the NLR for the diagnosis of AA and complicated cases [7,13,14,28,29,30,31]. However, none of them concomitantly investigated the potential use of the serum Na^+^ level and the NLR for the prediction of CAA. Moreover, it has not been shown whether their combined evaluation significantly increases the reliability of the diagnosis of CAA.

Thus, we investigated the potential use of the serum Na^+^ level and the NLR in distinguishing CAA and UCAA and evaluated whether the reliability of the diagnosis of CAA was increased by the use of these two variables together.

## 2. Materials and Methods

Ethics Committee Approval and Patient Consent: This research was carried out in compliance with the Declaration of Helsinki and approved by our local institutional research ethics committee (institutional scientific review board number: 2024/3/11). Informed consent was not necessary because of the retrospective design of this study.

Definitions: The classification of CAA was determined by surgical evidence of a perforated or gangrenous appendix and the presence of an intraabdominal periappendiceal abscess or peritonitis [32]. According to our institution’s laboratory normal serum Na^+^ levels, hyponatremia is defined as a serum sodium level of ≤135 mmol/L, and normonatremia is represented by a serum Na^+^ level that is >135 mmol/L and ≤145 mmol/L.

Study Design: This retrospective, cross-sectional, two-center study included all adult patients who were diagnosed with AA and underwent an open or laparoscopic appendectomy at the clinics of general surgery between 1 January 2016 and 31 December 2023. The study was developed according to the STROBE checklist included as Supplementary Material [33].

Data collection: The extracted data included demographic characteristics, such as age and sex; intraoperative, histological findings; and serum Na^+^ levels, white blood counts, and NLR values measured at the time of emergency department presentation.

Study Population: A total of 3091 patients with a histologically confirmed diagnosis of AA were evaluated. Four patients who were hospitalized and received medical treatment in different health centers, three patients with renal failure, and two patients receiving immunosuppressive agents due to solid cancer were excluded. Patients in complicated cases were enrolled using surgical documentation of intraoperative findings. Because of this reason, thirteen patients whose surgical notes were not available through the computer-based hospital information system were eliminated. Also, 3 patients younger than eighteen years old were excluded. Finally, 3066 patients were included in this study.

Outcome Definition: We aimed to determine whether the NLR and the serum Na^+^ level were able to differentiate between UCAA and CAA and evaluate whether the reliability of the diagnosis of CAA was increased by the use of these two variables together. The secondary objective was to establish the demographic features related to CAA.

Data analyses: Evaluations were carried out with SPSS Statistics for Windows, version 22 (IBM Corp., Armonk, NY, USA). The patients’ characteristics were expressed using descriptive statistics. The variables with a normal distribution were expressed as mean ± SD (standard deviation), and the non-parametric data were given as the median distribution. An independent sample t-test was used to compare UCAA and CAA for the parameters with a normal distribution, while the Mann–Whitney U test was used for parameters with a non-normal distribution. Multivariate logistic regression analyses were used to investigate the correlations of sex, age, the serum Na^+^ level, and the NLR with CAA. A receiver operating characteristic (ROC) curve was created to assess and compare the accuracies of the NLR and the serum Na^+^ level. The area under the curve (AUC) reflected the accuracy of the blood markers in identifying CAA and UCAA. For each biomarker, cutoff points, sensitivity, and specificity with 95% confidence intervals and a likelihood ratio were established. *p* < 0.05 was defined as being statistically significant. Cohen’s Kappa analysis was performed to quantify the level of agreement between the serum Na^+^ level and the NLR in diagnosing CAA.

## 3. Results

Of all the patients treated with appendectomy between 1 January 2016 and 31 December 2023, a total of 3099 patients who had a histologically confirmed case of acute appendicitis were detected and evaluated. Four patients who were hospitalized and received medical treatment in different health centers, three patients with renal failure, and two patients receiving immunosuppressive agents due to solid cancer were excluded. Thirteen patients whose surgical notes were not available through the computer-based hospital information system were eliminated. Also, three patients younger than eighteen years old were excluded. Finally, 3066 patients were included in this study. Most of the patients underwent open surgery, with laparoscopic appendicectomies representing 23.1% (709/2357). Among the cases included in this research, 1770 (57.8%) were male, and 1296 (42.2%) were female. The mean age was 39.4 ± 15.49 years.

According to the surgical notes, the patients were categorized into CAA and UCAA. The registered data revealed that 348 (11.3%) patients had CAA, and the remaining 2718 (88.7%) patients had UCAA. The patients with CAA were significantly older than those with UCAA (49.47 ± 18.97 vs. 38.16 ± 14.50; *p* < 0.001). The median serum Na+ value was significantly lower in the patients with CAA as compared with those in the cases of UCAA. The patients with CAA had a significantly higher median NLR. Additionally, there was a statistically significant difference between UCAA and CAA in terms of white blood cell count, absolute neutrophil count, and absolute lymphocyte count (Table 1).

A model including age, sex, the serum Na^+^ level, and the NLR was created. According to the Hosmer and Lemeshow chi-square goodness-of-fit test (*p* = 0.206), it was concluded that “the model represents the data well”. These variables were submitted to multivariate logistic analysis, and the evaluation revealed that age (odds ratio (OR): 1.03, 95% confidence interval (CI): 1.02–1.04), sex (OR: 1.37, 95% CI: 1.06–1.76), the NLR (OR:1.02, 95% CI: 1.01–1.03), and low serum Na^+^ (OR: 0.68, 95% CI: 0.68–0.75) levels correlated with the risk of CAA (Table 2).

**Figure 1:**
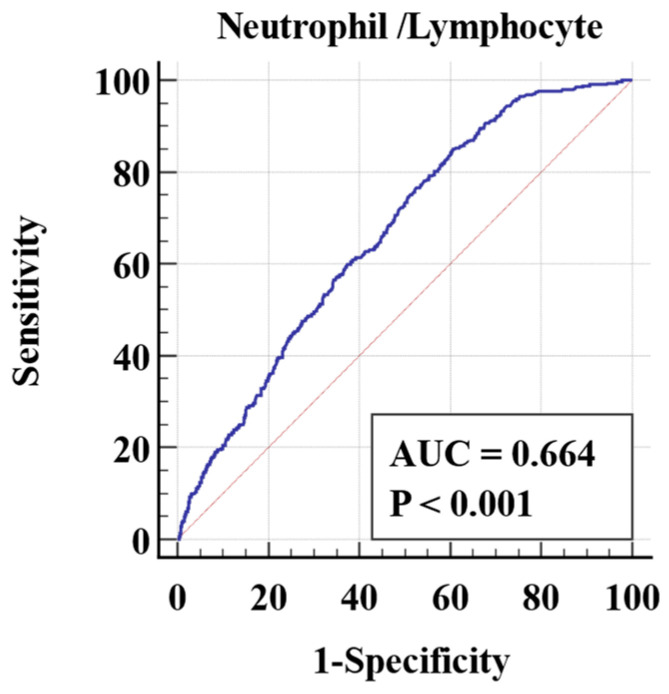
Analysis of receiver operating characteristic (ROC) curve of neutrophil-to-lymphocyte ratio in patients with complicated acute appendicitis.

**Figure 2:**
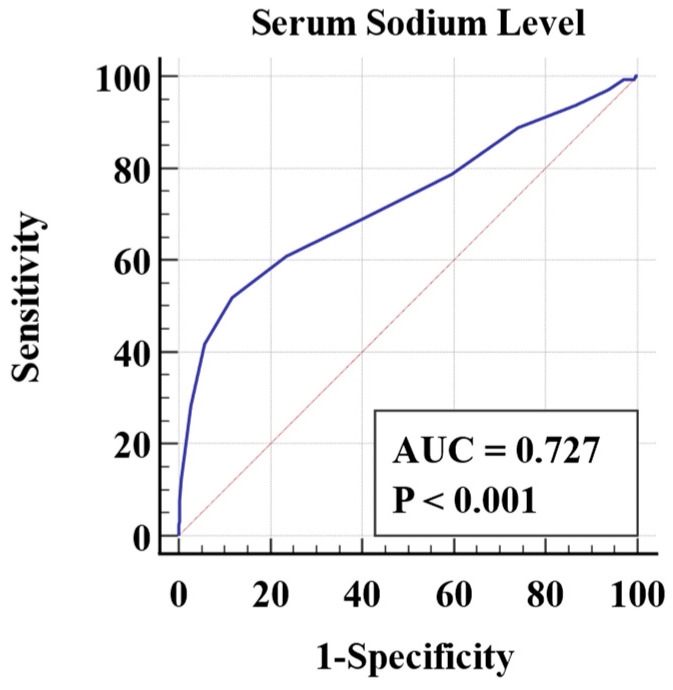
Analysis of receiver operating characteristic (ROC) curve of serum sodium level in patients with complicated acute appendicitis.

The AUCs of the NLR and the serum Na^+^ level are shown in Figure 1 and Figure 2, respectively.

The sensitivity and specificity of the NLR in predicting CAA at the cutoff point of 4.2 were 85.01% and 39.33%, respectively (95% CI: 0.647–0.681; *p* < 0.001). The likelihood ratio of having CAA compared with having UCAA was 1.39 times higher in the patients with an NLR of >4.2 (%95 CI: 1.32–1.47). The negative likelihood ratio was 0.39 (%95 CI: 0.31–0.51).

ROC curve analysis used a serum Na^+^ level of ≤135 mmol/L as the optimal cutoff point, with a sensitivity of 51.87% and a specificity of 88.34% (%95 CI: 0.711–0.742; *p* < 0.001). The likelihood ratio of having CAA compared with having UCAA was 4.45 times higher in patients with a serum Na^+^ level of ≤135 mmol/L (%95 CI: 3.85–5.14). The negative likelihood ratio was 0.54 (%95 CI: 0.49–0.61).

Cohen’s Kappa analysis revealed an exponential correlation between the NLR and the serum Na^+^ level, with a moderate degree of agreement in CAA (Cohen kappa 0.461, *p* < 0.001) [34].

## 4. Discussion

The diagnosis of acute appendicitis (AA) and the differentiation of complicated cases using blood markers remains a topic of interest in surgical research. The Alvarado and appendicitis inflammatory scores, which are well-known and widely used supplementary clinical tools in the diagnosis of AA, are considered to be inadequate in ruling out complicated cases [5,6]. The need for the development of a validated, easily accessible, and interpretable predictive model to differentiate CAA from uncomplicated cases has not been met. This study investigated the potential use of the serum Na^+^ level and the NLR in differentiating CAA and uncomplicated cases and evaluated whether the reliability of the diagnosis of CAA was increased by the use of these two variables together. The results may influence the choice to continue with surgery or to manage the patient using non-surgical treatment.

An increased NLR and hyponatremia have also been linked to several other acute conditions associated with intra-abdominal pathologies, such as intestinal ischemia in the small bowel ileus [35] and the severity of cholecystitis [36,37]. The potential mechanisms for hyponatremia and an elevated NLR in association with a significant inflammatory response have been explored in some studies [19,20,21,22]. The induction of a local inflammatory reaction causes an increase in growth factors, tumor necrosis factor-alfa (TNF-a), and interleukin-6 (IL-6). Growth factors facilitate neutrophil proliferation, while TNF-a mediates lymphocyte apoptosis. Generally, these are reflected in the overproduction of neutrophils and a reduction in lymphocytes [19,20]. Raised levels of circulating IL-6 activate the subfornical organ and organum vasculosum, and this leads to increased vasopressin secretion by neurons of the supraoptic, paraventricular nuclei. Hence, dilutional hyponatremia occurs as a result of cytokine-mediated non-osmotic vasopressin secretion [21,22].

In this retrospective study, a correlation between decreased serum Na^+^ levels, increased NLRs, and CAA was observed. Adults with perforated or gangrenous appendicitis had significantly lower serum Na^+^ levels and a higher NLR than those with UCAA. At the time of admission to the emergency department, a serum Na^+^ level of 135 mmol/L or less and an NLR of 4.2 or more were predictors of CAA. Multivariate analysis showed that each unit decrease in the serum Na^+^ level increased the rate of CAA by 0.722 times, while each unit increment in the NLR raised the rate of CAA by 1.02 times.

In a prospective study, Pogorelić and co-workers reported hyponatremia as a predictor of CAA, with a sensitivity of 94.7% and a specificity of 88.5% at a cutoff serum Na^+^ concentration of less than 136 mmol/L [25]. A similar study by Elgendy et al. was also able to reveal that a cutoff serum Na^+^ level of <135 mmol/L distinguished CAA, with a sensitivity of 94% and a specificity of 91% [27]. Retrospective research conducted by Messias et al. established a serum Na^+^ level of 136 mmol/L as the optimal cutoff point, and hyponatremia was correlated with the risk of CAA (OR: 0.74, 95% CI: 0.68–0.80). The sensitivity and specificity were 45.7% and 86.4%, respectively [26]. These results were quite similar to the findings of our study, with a sensitivity of 51.87% and a specificity of 88.34%. Additionally, some recently published meta-analyses investigated the role of hyponatremia as a diagnostic marker of CAA, in a population mainly consisting of pediatric patients. They suggest that hyponatremia may be useful in the diagnosis of CAA [8,23,24]. We also demonstrated that the NLR can make a distinction between simple and complicated appendicitis, with a sensitivity of 85.01% and a specificity of 39.33% at a cutoff point of 4.2. In a recently published study conducted by Esquivel et al., a cutoff point of 7.4 was estimated, with a sensitivity and specificity of 68.6% and 56.3%, respectively. Patients with an NLR of 7.4 were 3.8 times more likely to have CAA as compared with that of the simple cases [29]. Hajibandeh et al. were also able to establish a cutoff value of 8.8 for the NLR in the diagnosis of CAA. This threshold had a sensitivity of 76.92% and a specificity of 100% [7]. Additionally, as compared with the cutoff point of 4.2, Ayeni et al. and Prasetya et al. observed higher cutoff points for the NLR to distinguish the patients with CAA from the patients with UCAA [13,28]. However, our cutoff level of 4.2 was statistically significant, with an area under the ROC curve of 0.664 and a *p*-value of <0.001. One interpretation of this variation in cutoffs could be related to the fact that we used blood tests taken at the time of the presentation to the emergency department. In the meta-analysis conducted by Hajibandeh et al., only two out of the thirteen studies evaluating CAA clearly stated that the blood test results used in this study were the first to be performed in the emergency department. In five of them, the blood results were defined as laboratory tests performed at hospital admission [7]. In addition to the remaining studies in this meta-analysis, no information on this issue was available in the study conducted by Prasetya et al. [28]. Meanwhile, a study by Ayeni et al. included patients receiving either conservative or surgical management for AA [13]. Thus, there may be an increase in the NLR as an indicator of ongoing inflammation between the time of emergency department presentation and surgery [1]. An alternative probable interpretation for the higher cutoffs could be related to the age of the patients. CAA is more common in adult patients aged over 65 years and children aged less than 12 years [3,25]. These patients may have non-specific presentation symptoms and longer intervals between the onset of symptoms and hospital admission. The results of the studies including patients in these age groups support this alternative possible interpretation [7,13,25,29].

In our study, when the discrimination power of the NLR and hyponatremia in CAA was compared (AUC: 0.66 vs. 0.74, respectively), we concluded that the serum Na^+^ level was a more effective marker to distinguish complicated acute appendicitis from simple acute appendicitis. In the patients with CAA, there was an exponential correlation between the NLR and the serum Na^+^ level with moderate agreement (Cohen kappa 0.461, *p* < 0.001). This confirms that the combined evaluation of the NLR and the serum Na^+^ level significantly increases the accuracy of the diagnosis of CAA. The results support the choice to continue with surgery in patients who have an increased NLR and hyponatremia. Also, in view of the findings obtained, non-operative treatment with antibiotics may be an alternative option in cases of uncomplicated acute appendicitis.

Imaging modalities are usually used to confirm the diagnosis of AA. Additionally, they can be used to guide the differentiation of CAA and UCAA. CT is the preferred imaging modality for these goals. According to the data in a recently published systematic literature review including 15 studies, the estimates of sensitivity and specificity for CT varied from 28 to 95 and from 71 to 100 percent, respectively [11]. The mean sensitivity was 78% (95% CI: 64–88), and the mean specificity was 91% (95% CI: 85–99). When used as a part of the discussion about distinguishing CAA and UCAA, the sensitivity and specificity of CT are likely to vary. This shows that tomography is not a flawless tool in this practice.

A possible limitation of the current research is associated with its retrospective design. However, its validity is improved by the sample size and multicenter setting. Additionally, not all the variables that may be associated with complicated appendicitis were examined; nevertheless, the main aim of our study was to evaluate the easily accessible and regularly ordered variables used in the investigation of adult patients at the time of admission to an emergency department. The inability to evaluate the effect of the time interval between symptom onset and hospital admission due to the retrospective design of this study was another limitation of our study. Despite the fact that all the cases within the time frame were included in the study, and strict rules were used to allocate the patients to the case-control groups, the retrospective study design may have caused unintended bias in patient selection. Multicenter, prospective evaluations including standardized age groups and time intervals between symptom onset and surgery are needed to remove the inconsistencies with the cutoff values used for blood markers and to make comparisons more reliable.

## 5. Conclusions

The diagnosis of acute appendicitis represents a common challenge, with a postoperative period associated with high morbidity, especially for patients with surgical findings such as ischemia, perforation of the appendix, and local or generalized peritonitis. The serum Na^+^ level and the NLR may serve as cost-effective, easily accessible, and interpretable blood markers of CAA. Although the serum Na^+^ level is a more effective marker than the NLR, the use of these two variables together may detect high-risk patients who may benefit from early management in an effort to limit delays in surgery.

## Figures and Tables

**Table 1:** Analysis of clinical and demographic variables.

	Non-Complicated	Complicated	*p*-Value
Age (years) ^1^	38.16 ± 14.50	49.47 ± 18.97	<0.001 *
WBC (cell/mm^3^) ^2^	12.9 [10.1–15.7]	14.5 [11.1–17.8]	<0.001 *
Neutrophil (%) ^2^	77 [68.2–83.4]	81.5 [75.3–86.5]	<0.001 *
Lymphocyte (%) ^2^	14.8 [9.7–21.8]	10.6 [7.2–15.1]	<0.001 *
NLR ^2^	5.19 [3.11–8.48]	7.54 [5.06–11.85]	<0.001 *
Serum sodium level (mmol/L) ^2^	138 [137–140]	135 [133–138]	<0.001 *

^1^ Data are presented as mean ± standard deviation (SD). *p*: independent sample *t*-test; * *p*-value is significant at ≤0.05 level. ^2^ Data are shown as median [Q1–Q3]. Q1: first quarter value; Q3: third quarter value; *p*: Mann–Whitney U test; * *p*-value is significant at ≤0.05 level. White blood cell: WBC; neutrophil-to-lymphocyte ratio: NLR.

**Table 2:** Multivariate logistic model for complicated acute appendicitis.

	B	Wald	*p*-Value	Exp(B)	95% Confidence Interval for Exp(B)	
					Lower	Upper
Age	0.034	89.344	<0.001 *	1.035	1.028	1.042
Sex	0.317	6.026	0.014 *	1.373	1.066	1.769
NLR	0.023	13.364	<0.001 *	1.024	1.011	1.037
Serum sodium level	−0.326	185.158	<0.001 *	0.722	0.689	0.757

* *p*-value is significant at ≤0.05 level.

## Data Availability

The data presented in this study are available on request from the corresponding author due to legal restrictions.

## Supplementary Materials

The following supporting information can be downloaded at: https://www.mdpi.com/article/10.3390/medicina60111844/s1, The study was developed according to the STROBE checklist included as Supplementary Material.
